# Close, yet so far away: a phenomenology of the praecox feeling in the diagnosis of schizophrenia as intercorporeal alienness

**DOI:** 10.3389/fpsyt.2024.1445615

**Published:** 2024-10-02

**Authors:** Iván Vial, Marcin Moskalewicz, Anastazja Szuła, Michael A. Schwartz, Thomas Fuchs

**Affiliations:** ^1^ Phenomenological Psychopathology and Psychotherapy, Psychiatric Clinic, University of Heidelberg, Heidelberg, Germany; ^2^ Philosophy of Mental Health Unit, Department of Social Sciences and the Humanities, Poznan University of Medical Sciences, Poznań, Poland; ^3^ Institute of Philosophy, Marie Curie-Sklodowska University, Lublin, Poland; ^4^ IDEAS NCBR (National Centre for Research and Development), Warsaw, Poland; ^5^ Department of Psychiatry and Humanities in Medicine, Texas A&M Health Science Center, College, Station, TX, United States

**Keywords:** praecox feeling, phenomenological psychopathology, alienness, metaphor, clinical decision-making, diagnostic techniques and procedures, embodiment, intercorporeality

## Abstract

Debates concerning the reliability and validity of operationalized criteria and diagnostic tools have surrounded the issue of schizophrenia diagnosis and clinical decision-making related to the disorder. The notion of the praecox feeling (PF) has played a prominent role in the discussions as an example of the possibility of a rapid and potentially valid diagnosis based solely on “intuition” or a peculiar emotional experience or impression arising in a physician during an interaction with a patient with schizophrenia. In this paper, we argue that PF is enabled by the (phenomenologically understood) intercorporeal dimension of the clinical encounter. Intercorporeality in this sense denotes intertwinement between embodied expressions that may lead to feelings of connection but also, as in the case of PF, of disconnection and strangeness—the experience of alienness. Following Waldenfels, alienness ranges from the average social encounter to more extreme and peculiar forms—such as PF. To prove our point, we analyze the metaphors used by physicians in various cultural contexts (the United States, the United Kingdom, and Poland) to express the apparently ineffable experience of the PF. We focus on two dominant metaphors of distance: the first expressing spatial distance by referring to an “object in-between” the physician and the patient and the second expressing mental distance by referring to the “other-worldliness” of the patient. We interpret the object in-between metaphors as reflecting the sense of separateness and the other-worldliness metaphors as reflecting the sense of strangeness, with both meanings unified in the notion of “close remoteness.” Such unsettling but speculation-provoking feeling of close remoteness may be rendered by the concept of “the eerie” (Mark Fisher). We conclude that metaphor and phenomenological analysis facilitate an understanding of the experiential profile of PF in the clinical encounter, outlining relevant clinical implications.

## 1 Introduction: the issue of praecox feeling

The issue of psychiatric diagnosis and clinical decision-making regarding mental illness has always been surrounded by debates concerning its adequacy, possible implicit bias, the role of values and culture, and the specificity of psychiatry vis-a-vis other fields of medicine (1–10). These aspects are especially relevant regarding schizophrenia, one of the most debilitating and complex mental disorders to diagnose and treat. The debates concerning the reliability and validity of operationalized schizophrenia diagnosis are ongoing, with a number of new clinical tools both developed and validated in various cultural contexts (11–22). What is, however, mostly neglected in this debate—with a few notable exceptions, for example, the Assessment of Clinician’s Subjective Experience (ACSE) (23)—is the phenomenal content of clinicians’ subjective experience and their personal feelings and intuitions. However, the latter subject has long been explored in the European tradition of phenomenological psychopathology, in which personal attitudes and feelings were often discussed as a possible diagnostic compass with its own validity irreducible to the operationalized criteria (1, 24–32).

The most pronounced of these explorations concerned the notion of the “praecox feeling,” coined by Dutch psychiatrist H. C. Rümke and popularized through an article from 1941 (trans. 1990), “The nuclear symptom of schizophrenia and the praecox feeling” (33, 34). Rümke argues that a feeling experienced by the clinician in the encounter with a patient with schizophrenia is “the final and most important [diagnostic] guideline” [(34), p. 336]. Classical phenomenological psychiatrists like Eugène Minkowski and Ludwig Binswanger had already employed related terms such as “diagnosis by penetration” and “diagnosis with feeling” (35, 36). However, Rümke’s article had a particular resonance and motivated an intense debate on schizophrenia and the role of subjectivity in diagnosis. With time and the advent of an operationalized perspective on diagnostics, the debate surrounding praecox feeling gradually faded (27, 37). The controversy around the praecox feeling (henceforth, PF) has flourished in the last two decades. An important reason for this rebirth has been the appearance of empirical studies on the diagnostic reliability and specificity of PF (38–42).

Over and above the controversy around the diagnostic use of PF and the promising indications of recent empirical evidence, some structural questions still need to be addressed in depth. We wish to highlight two interrelated problems. The first set of questions concerns how the clinical encounter is framed. How do we conceptualize the embodied and affective aspects of clinical encounters? Can the encounter be the locus of feelings, and if yes, how is this possible? Can something like feeling strangeness, often adduced in PF research, be made sense of? Giving PF theoretical backup but disregarding it as a myth or an ill-conceived concept (27) depends on how one answers these questions.

The second set of questions relates to PF as such. A fundamental question that has rarely been explored is: What does one actually experience when feeling praecox? Can we specify the feeling(s) of praecox feeling? If yes, how? What tools can enable us to profile the subjective experience of PF? Exploring these latter questions is of crucial importance to the debate on PF. The question of whether PF indicates schizophrenia presupposes the question of whether there is an experiential structure specific to PF. In this sense, attempting to describe the experiential profile of PF is the only ground on which we can eventually (and possibly empirically) evaluate whether something qualifies as PF. By having a clearer picture of how PF feels and is lived through, its diagnostic use can be assessed in a different and more phenomenologically grounded light.

This article explores these two sets of questions from a phenomenological perspective. In Sections 2 and 3, a theoretical framework for grounding PF is offered. Section 2 proposes to grasp the embodied and affective dimension of the clinical encounter through the concept of intercorporeality. In Section 3, and as a preparatory analysis of PF, we describe and discuss the dimensions of identification, differentiation, and alienness that belong to intercorporeal experience. Afterward, in Section 4, an empirical analysis of metaphors on PF based on survey-based research in several countries and the results of the analysis of the PF metaphors is presented. Subsequently, Section 5 integrates the conclusions of the previous sections and the existing literature on PF. We propose that PF exhibits a phenomenological structure of “close remoteness.” Section 6 delinates clinical implications and offers future directions.

## 2 The intercorporeality of the clinical encounter

To underpin the phenomenology of PF, it is first crucial to thematize the context in which it appears, namely, the encounter between physician and patient. It corresponds to social interaction, i.e., a social situation where both parties are co-present and communicate with one another: the doctor experiences the patient, and the patient experiences the doctor, *as* they face and interact with each other. Negatively put: in the doctor–patient encounter, neither the doctor, the patient, nor the exchange can be removed from the picture. Unlike the situation where, say, the doctor is reading the medical records of the patient about to come or answering her email, the patient and doctor are each tied to an embodied “here” and “there” running in both directions (doctor to patient, patient to doctor).

Although the social interaction between doctor and patient can be pinned down by drawing upon diverse phenomenological concepts, *intercorporeality* is particularly pertinent to shed light on the issue of PF. Other phenomenologically inspired concepts that have been applied to PF are for example the concepts of “typification” (1), “Gestalt perception” (27), “I-Thou intersubjectivity” (43), and “aesthetic judgment” (44). The choice of intercorporeality, as it will be progressively shown in this article, is precisely because it concerns the bodily and affective dynamics arising in social interaction. What do we mean by intercorporeality?

During social interaction, an exchange of bodily expressions and impressions between one’s own body and the other’s body sets forth. Intercorporeality, a concept first introduced by Merleau-Ponty, describes a sphere of exchange or resonance between the bodily expressions (gazes, movements, gestures, speech, etc.) and the feelings (curiosity, shame, connection, detachment, etc.) experienced by the subjects while interacting (45–52). Against this background, the structure of intercorporeality in a dyadic situation can be regarded as threefold: 1) the own body, 2) the foreign body, and 3) the intersphere, the intertwinement between them (49). Intercorporeality entails a proper *inter* between own and foreign body, an in-betweenness irreducible to each body in isolation because an entanglement or intertwinement between them emerges. In other words, intercorporeality cannot be interpreted in Cartesian terms, namely, as an encounter between two enclosed mental worlds attempting to decipher each other’s hidden messages and emotions (51). The intercorporeal intertwinement is displayed in various forms and can be more or less salient. A rather manifest case, for instance, would be the experience of a person who is struggling to contain his anger at you:

the restrained, ‘pent up’ anger of a person is not only felt in their facial expression but even more in one’s unpleasant feeling of being affected and rejected (…) his increased bodily intensity is transferred to the counterpart in the rigid tension of his gaze or the twitching of his hands. You can feel the other person in your own body. [(47), p. 418, own translation)

In that vein, the awareness of intercorporeality is initially passive and pre-reflective: we come to experience shame or anger, and as we argue, PF through a *pathos*, something that happens and touches us, that befalls us (*Widerfahrnis*), that is not staged or produced by reflection (53). In other words, the primary experience of the bodily exchange with the other does not arise at the level of higher-order social cognitions, such as the reflections or analogical inferences a subject may engage in to grasp or decipher what is going on in the other’s head, “as when Sherlock Holmes infers the suspect’s motive for the crime” [(54), p. 32]. Such a pathic and pre-reflective structure of awareness makes the concept of intercorporeality (of the clinical encounter) suitable to grasp PF, widely described as something that usually arises in the first minutes of the interaction and it happens “passively; it cannot be instigated at will” [(27), p. 1125].

## 3 Intercorporeality between identification, difference, and alienness

Human intercorporeality oscillates between the poles of identification and differentiation of own and foreign body. Interacting with another human comes with the quality of being equal, someone like me, i.e., another person, instead of another life form or a mere object (53). This is a minimal and pre-reflective form of identification, one that Husserl designated as a “pairing association” [(55), p. 112]. The intercorporeal intertwinement can reach higher degrees of identification. We may experience the interaction as predominantly harmonious or fluid, as when we experience a sense of reciprocity, attunement, connectedness, or intimacy with the other. Some instances favoring this kind of experience would be friendly conversations, playing games, making music, sexual intercourse, etc. In these moments, the sense of identification with the other goes beyond a mere pairing of the bodies and leads, as it were, to their intertwining. Merleau-Ponty designated such heightened intercorporeal identification as “mimesis”: an “ensnaring of me by the other” [(46), p. 145].

Even in such social encounters where the difference with the other is de-emphasized, it is not something to be watered down and dissolved, but precisely what enables us to coherently speak of a sense of affective reciprocity or connection with a foreign bodily subject (56). As phenomenologists like Husserl, Lévinas, Waldenfels, or Zahavi have systematically argued, the otherness of the other—i.e., its alterity to myself—is unfathomable; it is what furnishes the experience of the other with the sense of being an experience of the alien (*Fremderfahrung*) rather than a mere experience of myself (55, 57–59). If the other were not alien to me, to feel bodily attuned or intimate with him/her would lead to a kind of “melting” or “fusion.”

The praecox feeling experience is to be located on the opposite end of intercorporeal mimesis. In PF, a heightened alterity or differentiation between own and foreign body appears, taking the form of an atmospheric sense of alienness or strangeness—which some authors have labeled as “bizarreness of contact” (24, 30, 60). This atmospheric alienness is adduced in a formulation of PF by Rümke: “In the encounter with the schizophrenic patient, the investigator feels a curious hesitation and a feeling of strangeness” [quoted in (30), p. 136].

Similarly, in a recent article, Sass and Feyaerts describe the strangeness at issue as “an interviewer’s gut feeling of not being in sync with or readily able to empathize with the person being treated, who may seem to inhabit a rather different world” [(61), p. 475]. The bizarreness of contact points to a crucial issue in the phenomenology of the PF: how is such an atmosphere of alienness/strangeness to be understood? Yet, on the road to answering this question, the “phenomenal signature” of alien experience must be clarified first. A reason for this is that between the structural alienness of others and the one of PF, there is a path to be bridged, and one not free of difficulties.

Something one often stumbles upon in topics like alterity/otherness and alienness/strangeness is that they are determined through negativities, i.e., as characterized by what they are not. “The foreigner,” writes Kristeva, “can only be defined in a negative fashion” [(62), p. 95]. Foreign cultures appear as such in contrast to one’s culture, foreign languages to one’s language, other bodies to one’s body, alter egos to one’s ego, etc. Thus, what is not one’s own, near or known, induces us to portray alienness as an epistemic deficit (63). Yet, this line of thought is misleading. Certainly, all others are alien to oneself in a certain way, but to experience the alienness of the other involves more than a deficit of something. The experience of not understanding another’s gestures or intentions, or the one of strangeness in the encounter with the patient with schizophrenia, indicates a positivity, an experience of something. Indeed, if we stick to a purely negative picture, we will not be able to describe the qualities of the experiencing alterity or the alien, and consequently, we forfeit any possibility of specifying the alienness at stake in PF. When alterity and alienness amount to pure negativity, speaking about a “feeling of strangeness” becomes nonsense.

Conscious of the pitfalls of such a negative view of the alien, Waldenfels conceptualizes the stamp of the experience of what is alien (in German *fremd*) along Husserl’s paradoxical formulation, namely, as “accessibility of the originally inaccessible” [(55), p. 114, translation modified]. Something is accessible—not despite—but rather in its inaccessibility (64). The alien announces itself in a paradoxical correlation of presence and absence, where something shows itself (*sich zeigen*) by withdrawing itself (*sich entziehen*) (65). A concrete example would be hearing a foreign language:

Anyone who hears someone speaking in a foreign language that they don’t speak themselves hears what they don’t understand and at the same time realizes that they don’t understand it. Something shows itself to them by withdrawing from them. [(63), p. 9, own translation]

Following Waldenfels, already in the prosaic experience of hearing a foreign language, the alien is not mere negativity but an experience of something that shows itself by withdrawing from ourselves. In this sense, the experience of alienness (*Fremdheit*) can be best captured as a *spectrum*. In average cases, alienness remains in the background of the encounter. For instance, we experience the structural alterity of the other when he or she resists or contradicts our opinions, and we are not surprised by this fact because it is a basic component of engaging in a social encounter. At other times, the withdrawal experienced may escalate, and thus, alienness comes to the fore as a patent otherness, foreignness, or strangeness. By studying the commonalities of the German *fremd* with Western languages like English, French, Spanish, Latin, and Greek, Waldenfels proposes a threefold-structured polysemy of the alien that correlates to different realms of alien experience, where each dimension or axis exhibits a corresponding contrast:

*Fremd* is firstly that which occurs outside of one’s own region as being exterior, in opposition to being interior (…) *Fremd* is secondly that which belongs to others, in contrast to one’s own (…) Thirdly, *fremd* is that which belongs to a different kind, which is uncanny, peculiar, strange, in contrast to the familiar. [(59), p. 71f]

Therefore, by defining the experience of the alien as something that shows itself by withdrawing, and by outlining three dimensions where alienness appears, Waldenfels’ phenomenology of the alien offers a path to bridge the gap between the ubiquitous and multifaceted differences between self and other and the specific alienness at stake in PF.

The question that remains is how we can specify the alienness in question through something as elusive as the “pre-reflective feel” of PF. This intricacy is mirrored in the controversy around the diagnostic use of PF, which has been a target of criticism for a long time and from different traditions in psychiatry. For positivists, PF is purely subjective and therefore lacks any scientific validity; for anti-psychiatrists, PF is an example of the arbitrariness and excessive power of psychiatric labeling (27, 66). Parnas concedes some kind of epistemological value to PF in schizophrenia research, yet sentences: “It is obvious that praecox-feeling, for several reasons, cannot belong to the diagnostic tools in clinical psychiatry” [(27), p. 1125].

As was stated in the introduction, the aim of this paper is not to overcome these criticisms by concentrating on the problem of the factual diagnostic use of PF. Nonetheless, we propose that an important step to do so is to frame this problem along another problem, namely, the one between feeling experience and linguistic expression. If we want to clarify feelings, we must not only live them through but also reflect and thematize them. Otherwise, any such pathic experience becomes too vague and we are blind to the relevant nuances (67). This is crucial for the problem of the diagnostic use of the PF: if we do not take distance from the feeling, reflect, compare, and attempt to put it into words, we cannot fully know it. By leaving it in its pure “feel,” we run the danger of a certain “alexithymia.”

## 4 Metaphors of the praecox feeling

The following analysis is based on data from four samples, both historical and contemporary, where the Sagi and Schwartz questionnaire (with slight modifications) was applied (68). We focus on the answers to the open question only, which was asked to psychiatrists who declared occasionally experiencing “feelings” about a patient strongly suggestive of the diagnosis of schizophrenia and, simultaneously, were able to articulate these “feelings” in words. Except for the recent study of the Polish sample, these qualitative data have never been analyzed, while the Polish sample was not researched regarding the metaphors used. The quantifiable results of the survey were published regarding two of these samples, 1989 New York (68) and 2019/20 Poland (42), while the results of the 2017 New York and the 2018 UK studies remained unpublished. The samples differ in type: the 1989 New York sample was randomized (*N* = 257), while the 2017 New York (*N* = 36), 2018 UK (*N* = 93), and 2019/20 Poland (*N* = 243) were convenience samples. The total number of participants is 629. The total number of respondents who experienced PF across the samples was 526 (83.62%). Out of those 526 psychiatrists, 335 (i.e., 63.68%) declared to be able to express PF in words and then filled an appropriate survey slot with a qualitative description, in particular, 164 from the 1989 New York study, 21 from the 2017 New York study, 47 from the 2018 UK study, and 103 from the 2019/20 Poland study [for more details regarding the numbers in published studies, see (42, 68, 69)]. These 335 qualitative descriptions were the object of further analysis.

Putting something like the PF into words is certainly not easy, but an experience that is precluded from linguistic expression—and therefore also of intersubjective validation—cannot become a reliable diagnostic tool either. In this regard, as Varga argues, Hempel’s positivistic criticism of PF is on the mark (43). Yet, rather than simply invalidating PF as a diagnostic tool, this intricacy also calls for medical education in phenomenology (70). A productive way through which surveyed physicians confront the problem of “ineffability” is the employment of metaphors. Complex and elusive phenomena like PF do not let themselves be expressed so easily, but tools like metaphorical language indeed allow us to point out and depict experiences and communicate them. In this sense, our thrust is that an analysis of the metaphors employed in PF profiles essential aspects of its phenomenology, which in turn enables its individuation.

If classic emotions such as anger, fear, or joy already present difficulties for verbal expression, PF lacks a literal expression altogether. Thinking in figurative language comes in handy. Rivers of ink have been spilled over metaphor, and it is beyond the scope of this article to go into exhaustive detail. We will follow Hanna Arendt’s view of metaphor (71). Conceived since Aristotle as a transfer between domains, Arendt argues that the metaphorical transfer bridges non-sensory thought and embodied sensory experience: “the mind’s language by means of metaphor returns to the world of visibilities to illuminate and elaborate further what cannot be seen but can be said” [(71), p. 109]. In this way, metaphors can be particularly appropriate for profiling the experience of PF: although making a metaphor requires thinking or reflecting, its particular form of figuration does not fly away into the realm of the conceptual or abstract but is anchored in the concrete and bodily felt realm of experience (72, 73).

In his study of metaphors regarding friendship, Kövecses argues that the difficult-to-grasp idea of an emotional relationship can be understood and communicated in terms of distance between two entities—the more intimate the relationship, the shorter the distance, hence intimacy is understood as closeness (74). The opposite, lack of intimacy—unfamiliarity, strangeness—is often referred to as distance. Most of the clinicians who participated in all studies spontaneously used metaphorical language in their descriptions of PF. They often used common expressions that metaphorically describe unfamiliarity in terms of distance, such as detachment, unconnectedness, remoteness, unrelatedness, (emotional) withdrawal, and out of touch. Some participants used unconventional metaphors, traditionally classified as alive or poetic (as opposed to dead conventional metaphors). In some descriptions, explicit metaphorical comparisons are present, where the source of the metaphorical mapping is introduced directly in the text—the metaphors are direct, as categorized by Steen (75), for example in the description that says that the patient “is like a ‘visitor from another planet’”.

Two major themes emerge. The first theme refers to an “object situated between” the actors that makes it virtually impossible to get through to each other, where the distance can be so great that the other person is physically not present (distance may be extreme, unpassable). Thus, the first theme recurs to a specifically “spatial” sense of distance. The second theme refers to a jarring difference with the other person—the contact being impossible because they are not the same (in extreme cases, not entirely human). This theme is depicted as an “other-worldliness” of the patient. Thus, the second theme emphasizes a more “mental” sense of distance. The two senses of distance (spatial and mental) overlap in some cases, e.g., “visitor from another planet.” With all conventional metaphors set aside, we summarized the frequency with which the two major themes appear in a direct manner in all 335 descriptions of the PF. Unconventional metaphors appeared in 72 (21.49%) descriptions, with metaphors of distance in 41 descriptions (12.24% of all descriptions, 56.94% of descriptions that contain a direct metaphor). We distinguished two major subcategories of unconventional metaphors of distance, termed “object in-between” and “other-worldliness” (present in 29 descriptions, 40.28% of descriptions with direct metaphors). **Table 1** presents the frequencies and examples of these metaphors in all four samples.

**Table 1:** Praecox feeling expressed through unconventional metaphors of distance.

Object in-between	*N* = 15 (20.83% of descriptions with direct metaphors)
New York 1989	
#72: *Patient makes me feel he/she is distant, disconnected and illogical -* ***there is a barrier***	
#82: *Dependent upon where the patient is or how they are feeling as to the intensity - and cause of illness:* ***interpersonal “space” and walled distance*** *; patient’s heightened sensitivity to therapist’s mood and general affective state*	
#100: *A sense of “strangeness”, guardedness, disconnectedness and internal preoccupation but more than “paranoic” guardedness - “otherness”*, ***a chasm*** *, (painful) separateness and aloneness*	
#106: *Described years ago as* ***“invisible wall”*** *between patient and doctor and referring to patient’s difficulty in connecting to others*	
New York 2017	
#27: *Experience a flatness, almost* ***like a wall is up between me and the person***	
United Kingdom 2018	
#28: *There is often an appearance of* ***being behind a glass wall*** *, of not being in the same room as the patient*	
#44*: I’m a great believer in the ‘praecox feeling’ of Rumpke - an impression of an* ***emotional barrier*** *, the patient presenting an aura of detachment and inner preoccupation*	
#56: *Ultimate impossibility to connect, as if there were an* ***obstacle in between***	
#62: ***Pane of glass*** *between me and the patient*	
Poland 2019/2020	
#60: *The patient is as if in another place, absent, distant, cut off from his feelings*, ***“behind a glass”***	
#111: *Patients seem to be sort of* ***“behind a glass”*** *, no emotional contact*	
#114: *Feeling that the patient is* ***“behind a glass”*** *, emotional rigidity*	
#157: *Contact with the patient* ***behind a glass*** *, poor emotionality*	
#172: *Even in the case of symptom dissimulation, the patient appears to the examiner as a person with a different sensory perception, the contact seems to be* ***“from behind a glass”*** *. The gaze of the patient also tells the examiner a lot, not without reason we call the eyes the mirror of the soul. Psychotics often hide right there. Another point which, in my opinion, is strongly underestimated is the patient’s characteristic syntax of speech, the way it is constructed and the choice of words.*	
#179: *Affective pallor, poor facial expression or amimic face*, ***patient remaining “behind a glass”*** *, rigid, restricted contact. Lack of eye contact or staring at the interviewer, insistent eye contact, gaze suggestive of hallucinations, concentration problems, deferral of responses, delusions, psychotic anxiety. A certain strangeness felt during the examination*	
Other-worldliness	*N* = 14 (19.44% of descriptions with direct metaphors)
New York 1989	
#18: *A feeling of extreme alienation*, ***living in a world distant from mine and others***	
#37: *That “praecox feeling” - a sense of* ***unreality*** *, remoteness, dazedness, that initially pervades my interview with the patient*	
#46: *Evidence of symbiotic mode of relating [no boundary between self and patient (or interviewer)] pt is like a* ***“visitor from another planet”*** *feeling of unconnectedness, either distance or negativism*	
#62: ***An “out-of-this world” quality*** *, i.e., an unrealistic view of the environment*	
#88: *Patient’s thought processes are functioning in a* ***“nonlinear” universe*** *; it feels as if they are* ***“disconnected” from the “normal” world***	
#190: ***It is a sense of other-worldliness that the patients are at the time of meeting in different worlds***	
#228: ***The patient is not on this planet*** *, projects a great deal*	
#233: *An eery feeling that the person is not in contact [ … ]. Prefers or is unable not to* ***“live in a dream”***	
#238: Feeling that the patient is **“out of this world”**	
#240: That the patient is somehow not connected to the agenda of the interview, that in subtle (or not so subtle) ways, **they are blocked off the world**	
United Kingdom 2018	
#47: *It is a sense of* ***other-worldliness*** *that the patients are at the time of meeting* ***in different worlds***	
Poland 2019/2020	
#15: *A sense of disorientation, distance, confusion. A feeling of following* ***the patient “drifting away”*** *, or a feeling as if* ***the voice is “coming” from a distance*** *(metaphor)*	
#88: *Inadequacy of affect and thinking*, ***“detachment from reality”*** *of the patient*	
#182: ***Being next to reality*** *, [ … ] and many other indescribable*	

Source: own study. Bold added by us. Own translations from Polish into English.

Finally, diverse terms belonging to the semantic field of the concept “alien” were also mentioned frequently in all samples. From the total 335 descriptions, and excluding direct references to psychiatric symptomatology (e.g., affect and thought disturbances), the 10 most frequent terms related to alienness are “bizarre” (*N* = 29, 8.66%), “strange” (*N* = 27, 8.06%), “odd” (*N* = 22, 6.57%), “distance” (*N* = 22, 6.57%), “different” (*N* = 19, 5.67%), “inadequate” (*N* = 19, 5.67%), “inappropriate” (*N* = 14, 4.18%), “unrelated” (*N* = 12, 3.58%), “detachment” (*N* = 11, 3.28%), and “unusual” (*N* = 10, 2.99%).

## 5 The intercorporeal alienness of the praecox feeling

In this section, we aim to generate a discussion that integrates the findings on the metaphors employed by physicians to describe PF, existing literature on the topic, and the framework of intercorporeality and alienness so far developed.

### 5.1 Close remoteness: separateness and strangeness

A special kind of correlation between presence and absence arises. Along the paradoxical language of the alien, we propose that the withdrawal at stake in the PF takes the general form of a *close remoteness*: of a multifaceted distance that appears when getting close to the patient. Contrary to the case of two people in love who, despite physical distance, feel near to each other, in PF, we are dealing with a remoteness *in* closeness. As one physician succinctly writes, the close remoteness of PF corresponds to a “feeling of strangeness and distance” (#NY 1989: 57). Nothing in the empirical studies on PF indicates that the pervading alienness felt in the interaction anchors to cultural or ethnic alienness. Rather, the patient is commonly experienced by the physician as remote in the sense of someone who is not there, distant, confused, or elsewhere. The patient is physically–spatially there, but not *co-present* in the intercorporeal space; thus, he appears to be left out or carved out of the *continuity of lived space* (51, 76). Expressed in Heidegger’s terms: his/her *Mitsein* (Being-with) is lacking, what remains is only *Vorhandensein* (Being-present-at-hand) in the physical space (77). Reversing Wim Wenders’ (1993) movie title *Faraway, So Close!*, here we say: close, yet so far away!

A particular aspect of the alienness that appears in PF is the impression of spatial *separateness*. Along with Waldenfels’ threefold dimensionality of alien experience, separateness refers to the contrast between interiority and exteriority, which usually corresponds to a contrast of place (59). Yet, the exteriority at stake in PF is not one of a distant place, but a remoteness *in* closeness. This paradoxical sense of separateness and absence that appears inside one’s proximal spatial region is what the metaphor of an object in-between describes: as if the patient were “behind a glass” or as if there were an “invisible wall” between them. The metaphor of the object in-between thus does not portray the physician or the patient as individuals, but rather, an interactional atmosphere of separateness, i.e., an impression arising from the in-between or the intersphere of intercorporeality.

The intercorporeal sense of separateness impacts the physicians’ experience of the relationship. Several authors have captured PF through interactional impasses, with concepts such as a lack of understandability, reciprocity, and empathy, to name a few (34, 78–80). Without denying that there is an obvious negativity or withdrawal at stake, we think it is better to avoid formulations that suggest a deficit. The withdrawal arises out of the intercorporeal exchange. For this purpose, we suggest “broken identification”: the physician engages in an empathic or attuning intention with the patient, but the attempt backfires. Before Rümke coined the term praecox feeling, Ludwig Binswanger described in 1924 such a broken moment of identification as an “inward rebound” (*innerlich zurückprallen*):

What we call a lack of rapport can, under certain circumstances, be the only perception I have of a stranger, but it can nevertheless be so ‘striking’ that I, so to speak, rebound inside myself, when the door opens and he enters (…) A schizophrenic patient can be very sympathetic to me as a human being, and yet I always rebound inwardly, I always have the impression that there is a barrier that prevents me from uniting myself deeply with him. [(35), p. 427, own translation]

Binswanger’s description of the separateness and the broken moment of identification adduces the metaphor of the object in-between, “a barrier that prevents me from uniting myself deeply with him.” If one follows Binswanger’s idea of the impossibility of unification, separateness obstructs intercorporeal identification on its high endpoint, which Merleau-Ponty calls mimesis. Feelings of attunement, connectedness, or intimacy seem to be forfeited by the “invisible wall.” Last but not least, Binswanger’s notion of “inward rebound” is particularly intercorporeal since it describes the interplay between the experience of the relation and the changes it involves in the physician’s self-experience. Varga (43) makes a similar point by alluding to Rümke when he says that in PF the investigator “notices something out of the order within himself” [ (34), p. 336]. Intercorporeal resonance is not approaching its nullification, but it is becoming alien.

Another particular aspect of the alienness that appears in PF is the recurrent impression of *strangeness*. How are we to understand this strangeness? One could think of the strangeness of the foreigner, which points to a cultural or national alienness, also part of Waldenfels’ topographic axis between the interior and the exterior. But this does not seem to be the case. In Waldenfels’ scheme, the referred strangeness rather unfolds on the axis that contrasts the familiar with the strange, the usual with the peculiar, “that which belongs to a different kind, which is uncanny, peculiar, strange, in contrast to the familiar” [(59), p. 72]. Strangeness as the peculiar, uncommon, or weird is the kind of alienness expressed by the metaphor of “other-worldliness”: “Out-of-this-world quality” (#NY 1989: 62), “Not on this planet” (#NY 1989: 228), or even like “A visitor from another planet” (#NY 1989: 46).

Let us draw some contrasts between the two metaphors. Compared to the metaphor of the object in-between, other-worldliness brings a more intense anchorage on the patient. Other-worldliness is a metaphor for the patient. However, it is also a metaphor that only takes that form because the physician locates him/herself in “this” world. In Husserl’s terms, there is a contrast between the “homeworld” (*Heimwelt*) and the “alien world” (*Fremdwelt*) at play (81). Like in the object in-between, there is a remoteness, but this time, the chasm or gap is much more radical, namely, the impression that the patient is, so to speak, a stranger in this world. The figure of the alien takes the shape of a science fiction-type alien, an extraterrestrial. This metaphor is connected with non-metaphorical strangeness. Again with Husserl, in its reference to an alien world, experiencing the strange (*fremdartig*) is itself the experience of a chasm, it is given as “accessibility in genuine inaccessibility, in the mode of incomprehensibility” [(81), p. 631, own translation]. Then, one can observe that whereas the object in-between metaphor represents a break in the high endpoint of intercorporeal identification (mimesis or intertwining), the other-worldliness metaphor expresses a shaking of ground at the low endpoint of identification, namely, the other as basically “someone like me” (pairing). To be sure, other-worldliness is a metaphor, a conditional “as if” that allows him/her to bring what seems ineffable into language. A broken sense of identification is at play, but after all, it is still a perception of the patient as a human being.

It is worth mentioning that the metaphor of other-worldliness echoes one of Rümke’s theses, namely, that through PF one can grasp the patient’s alienation from intersubjectivity outside the clinic. In his words, “the schizophrenic is outside the human community” [(34), p. 336]. However, Varga makes a critical comment on this idea: “From the concrete distorted encounter, one could argue, there is no way of inferring the complete alienation of the patient from the broadest sense of intersubjectivity” [(43), p. 140]. Rümke’s “outside the human community” is excessively strong and potentially derogatory. One should rather take it as a metaphorical expression that conveys the interactive strangeness felt while being close to the patient. This leads to another point. Rümke’s phrase is from 1941, and one of our samples is from 1989—where, for example, 10 out of 14 of the other-worldliness metaphors appear. Time has passed, and we are now much more aware of the derogatory power of linguistic expressions. In this sense, it is important to emphasize that we distance ourselves from any demeaning connotation when employing the concepts of alien or alienness in a phenomenological sense. Waldenfels’ phenomenological concept of the alien is not a pejorative label for others, but a spectrum of experience in which something reveals itself by withdrawing itself. In fact, alienness also concerns oneself. For instance, “my brain,” which realizes a large part of my experience, is nevertheless completely withdrawn from direct experience [see (65), p. 413].

### 5.2 The sting of the alien: feelings and praecox feeling

Up to this point, and through what we call close remoteness, we have situated the affective quality of PF mostly in the intercorporeal in-between. This is in line with other works on PF. As Varga argues, PF is not a secondary manifestation that appears through the physician’s frustration over the lack of connectedness, but “simply the manifestation of disconnectedness” [(43), p. 140]. Similarly, Moskalewicz and Gozé claim that PF “is not so much an impossibility of affective exchange as its bizarreness” [(30), p. 147]. These are crucial points: intercorporeal alienness is not just a lack of something, but a type of interactional experience that refers to an “incarnated absence” (*leibhaftige Abwesenheit*) [(63), p. 26].

The arousal of such a sense of absence may be linked with experimental findings on the behavioral correlates of schizophrenia at the level of interpersonal interaction. The mimesis or intertwining of bodies also has a rhythmic, temporal form. The moment-to-moment attunement of speech (e.g., prosody and turn-taking) and non-verbal expressions (e.g., movements and gestures) appearing in daily social interaction, ranging from delayed imitation to zero-lag synchrony, has been operationalized in behavioral terms as *interpersonal synchrony* or *coordination* (82–84). A significant impairment of non-verbal synchrony in schizophrenia affects both the patient’s and the interactant’s social behavior (85–92). A study of head movement synchrony showed how non-verbal synchrony varies with symptomatology (89): patients with higher scores on negative symptoms did not imitate their interactant, whereas patients with higher scores on positive symptoms were not imitated by their interactant. Impairments in speech coordination were also found (93, 94). A recent study (94) on turn-taking coordination shows that patients’ speech tends to overlap with the interlocutor more than controls and that negative symptomatology significantly correlates to periods of mutual silence. Finally, regarding emotional contagion (95, 96), which is an integral aspect of the affective dynamics of intercorporeality, a study (97) showed that schizophrenic patients exhibit diminished contagion of the other’s yawning and laughing.

The affective or emotional aspects of PF that relate to the patient and the clinician as bodily subjects are also critical. In his classical article on PF, Rümke (1941/1990) gave special relevance to the role played by the “affective disturbance” of the patient (33, 34). This intuition was empirically confirmed in Grube’s study (38). It was reported that the variable “affective disturbance,” compared to thought, action, and communication disturbances, had the highest impact on the intensity of PF. A grounded-theory-based analysis of the Polish 2019/20 sample (69) shows that the most predominant localization of PF is the patient’s affect or emotion (44%), most often described as emotional coldness (50%) and emotional rigidity and inappropriateness (26.09%). An above-quoted metaphor from the New York 2017 sample depicts quite well how the impression of separateness and noticing the patient’s emotion are correlative: “Experience a flatness, almost like a wall is up between me and the person” (NY 2017 #27). In this way, the intercorporeal atmosphere of separateness is likely to be correlated with the resonance that “flat affect” produces, a long-described negative symptom of schizophrenia (98). The impairments in facial expression of emotion and non-verbal communication during social interaction are well-known features of schizophrenia (99–104). That said, deficits in emotional expression and communication are not the whole story. In the study by Szuła et al. (69), 26% of the psychiatrists also attached a sense of unease to the patient, “A sense that the patient is confused, lost, feeling uncomfortable and anxious, or even threatened” [(69), p. 11, figure 2]. Finally, it is also worth mentioning that 55% of the respondents referred to the patient’s gaze, describing it as absent, blank, evasive, or distracted (69). Anomalies of gaze in schizophrenia patients during social interaction have also been consistently reported in behavioral studies (105–108).

On the side of the physician, PF deserves to be called a feeling also because it is endowed with an affective valence. The PF certainly comes nearer to the second between the classic poles of pleasure and displeasure, implying a pervasive feeling of *unease*. The experience of PF is indeed not comfortable or indifferent but may rather be anxiety-provoking, confusing, or disturbing (30, 69). Rümke’s late account of what he considers the core of PF also goes in the direction of a pervasive unease, namely, as an “inner insecurity” (*innerliche Unsicherheit*), and claims that it disappears with the aging of the patients (37, 109). Following Waldenfels, we come to experience the alien with a particular affective profile, as a “sting,” that “not only sets things in motion but also penetrates one’s flesh like the sting of a biting fly” [(110), p. 8, own translation]. Thus, experiencing something as alien does not only impact us, but it invades us, provoking turmoil in our body. Yet, how do we grasp the specific quality of the “sting” affecting the physician? An intricate aspect of this question is that such an unsettling feeling culminates in the diagnostic intuition that the patient suffers from schizophrenia. How does this happen? How does the feeling of close remoteness become the intuition of schizophrenia?

We propose to grasp the unease of close remoteness and the diagnostic intuition involved in the PF by drawing on Mark Fisher’s work on “the eerie” (111). His work on the eerie focuses on fiction in literature and cinema and, to our knowledge, has not been applied to psychopathology. Differences can be expected. The affection of the eerie concerns the unknown or the unknowable, a withdrawal of the alien that defies or even debunks our conceptual frameworks and familiar expectations. For Fisher, the tension between presence and absence is at its core. The eerie appears either as a *failure of presence*, i.e., “nothing is present when there should be something,” or as a *failure of absence*, i.e., “something is present when there should be nothing” [(111), p. 61). Fisher claims that the eerie involves a particular moment of suspense, curiosity, and speculation, where the central enigma concerns the unknown *agency* that should not be absent or present. Faced with the rocks of Stonehenge or Easter Island, there is a failure of presence; someone is missing, and we ask ourselves what kind of being could have crafted them. Hearing the cry of a bird can trigger an eerie failure of absence; we feel that there is an emotional intention at work that goes beyond mere reflex. Speculation becomes inevitable.

In the encounter with the patient, our expectations of how a social interaction flows are not met. At first glance, the PF is eerie in the sense of a failure of presence. In PF, the physician senses an “incarnated absence” that manifests as a broken interbodily identification, a lack of affective and kinetic attunement between him/her and the patient: “An eery feeling that the person is not in contact [ … ] Prefers or is unable not to live in a dream” (#NY 1989: 233). This eerie failure of social co-presence is not far from the classical notion of PF as a failure of empathy. However, when one takes the object in-between and other-worldliness metaphors of PF into account, what is brought into concrete imagery is a failure of absence: there is a wall between us, an alien world infiltrates the homeworld, something else is present where there should be nothing. Without paying attention to the metaphors of PF, this critical reference to the failure of absence may remain unnoticed. The close remoteness of PF resists a binary interpretation of the eerie, resulting in a hyperbolic eeriness where the expected is absent, and the alien layers itself over this void. Along with this remarkable eeriness, it is understandable that the unease of PF becomes extreme. As one physician wrote: “The person awakens an ‘uncanny’ feeling in me, along with a feeling of separateness, ‘weirdness’, otherness, and an uncomfortable constant anxiety” (#NY 1989: 16).

The unsettling eeriness of PF, however, does not freeze cognition but rather awakens speculation about the unknown agency pervading interaction, a tension that ends in the assertion that schizophrenia is such an agency. For an experienced clinician, a lengthy explicit process of speculation might not be required—which as one of us argued, might result in an intuitive typification (1). The interbodily encounter with schizophrenia is not wholly unfamiliar or unknown, it resembles past bodily interactions, so the clinician’s body memory (112) is a source of intuitive knowledge that resolves the suspense of PF with the schizophrenia diagnosis more quickly than a young trainee would be able to. Therefore, the PF as a “gut” diagnosis is not simply a feature of the actual intercorporeality but a cognition that is driven by the felt eeriness of the encounter and mediated by the physician’s embodied memory.

## 6 Clinical implications and future research on the praecox feeling

The foregoing analysis has several relevant clinical implications and opens venues for future research on PF, which we present below.

### 6.1 Clinical implications

#### 6.1.1 Intercorporeality and diagnosis

The physician’s intercorporeal experience is a crucial source of information about what is going on in the clinical encounter and the patient. Understanding PF as an intercorporeal alienness with the specific structure of “close remoteness” may theoretically enhance early schizophrenia diagnosis. Furthermore, although we have focused on PF and schizophrenia, a thorough analysis of intercorporeality in the clinic (totally neglected by the operationalized turn in psychiatric diagnostics) suggests the notion of *intercorporeal diagnosis* (52). For example, the ACSE tool (23, 113–115) has made crucial advances in this direction, investigating the experiential-affective features (i.e., tension, attunement, engagement, disconfirmation, and impotence) of the encounters with patients suffering not only from schizophrenia but also from mood and personality disorders, with their corresponding overall profiles.

#### 6.1.2 The alien as a psychopathological subject

The alien has been largely neglected as a theme of psychopathology. Adjectives like detached, disconnected, alienated, different, awkward, or strange are axiomatically taken as the downside of normality, mixed, and controversial and, therefore, scarcely thematized as such. The alien, however, is not a derogatory term in any sense, but a large and nuanced spectrum of phenomena that is constantly in dialogue with the sphere of oneself. As such, it is an essential component of the encounter with mental illness that second-person approaches to psychopathology (29, 114, 116–118) may progressively pin down and integrate also beyond schizophrenia. Developing mixed-methods research tools on intersubjective experience, like ACSE, is well-suited for this enterprise.

#### 6.1.3 Psychotherapy of schizophrenia

The present phenomenology of PF has implications for psychotherapy. Gozé (60) argued that the “bizarreness of contact” with schizophrenia, an intercorporeal alienness that is not the full-fledged PF, is also experienced by laymen. With this parallel in mind, therapists can develop more effective interventions regarding the patient’s struggles with intersubjectivity (79). An example would be to analyze the dynamics between the patient’s self-initiated social avoidance or withdrawal and the external avoidance or rejection they face due to intercorporeal alienness. Another suggestion is to enact containing bodily responses when alienness escalates during the session, allowing the emergence of “corrective relational experiences” through the interbodily dimension of the therapeutic relationship (52).

#### 6.1.4 Clinical training

None of the above clinical implications of our account of PF can be successfully integrated into clinical practice without education in its phenomenology (70). Concretely, the development of training programs for clinicians that include education on phenomenological features of the clinical encounter (such as intercorporeality and the nuances in the experience of the alien, such as difference, broken identification, distance, strangeness, and the eerie) is critical for the effective recognition of PF and its use in diagnostic and therapeutic contexts.

### 6.2 Future research on the praecox feeling

#### 6.2.1 Methodological improvements

First, a limitation of the studies we draw upon in our metaphor analysis is that they are based on a questionnaire (68) with just one open question about how PF is experienced. From the perspective of phenomenological research, it would be desirable to conduct microphenomenological interviews (119) on PF, which would allow for an in-depth exploration of its subjective experience. Second, all existent empirical studies on PF are based on reports given by physicians, and therefore, any comparisons with other non-medical professionals (such as therapists) or laymen are not possible. Although Gozé (60) suggests a parallelism between physicians and laymen regarding the bizarreness of contact, this comparison cannot be drawn precisely. Third, even if the construct of close remoteness becomes gradually vindicated, the inaugural diagnostic intricacy of PF remains: one thing is to say that there is such a thing as an experiential profile of PF, and another is that such a constellation of feelings is a reliable suggestion of the diagnosis of schizophrenia. Future studies on this topic could employ mixed methods that combine qualitative phenomenological interviews with experimental methods that measure the reliability and specificity of PF across diverse populations and contexts. With these tools in hand, the actual *validity* of the PF as a diagnostic tool could be assessed.

#### 6.2.2 The other side: the experience of the patient

There is not only one side of intercorporeality. We have shown that a critical component of PF is related to the patient’s expressions, but the patient’s experience of facing the physician remains highly understudied. The issue of experience is especially relevant since patients with schizophrenia show an incongruence between affective expression and experience: “I am hard as ice and yet so full of feeling that I am almost sentimental” [quoted in (120), p. 130)]. A predominant feeling that intrudes social interactions is their anxiety of “being other” (*Anderssein*) (61, 121–123) of being radically different and apart: “They are in one world, and I am in another” [(123) p. 1408]. In this vein, Sass and Feyaerts claim that the patient’s anxiety about being other “is the inner complement of the *praecox*-feeling” [(61), p. 479]. These suggestions certainly lead in an interesting direction, matching what we have described on the physician’s side. However, the specific “inner complement” of PF must be studied as a topic of its own, inquiring about the concrete otherness of the physician in the clinical setting, which may mismatch with PF. Qualitative phenomenological studies on the “other side” of PF can provide critical information about how patients respond to the clinician’s bodily expressions and intuitive assessments and their impact on the therapeutic relationship.

## 7 Conclusion

We proposed to address two sets of questions that remain highly untouched regarding praecox feeling from a phenomenological perspective. On the one hand, we aimed to make theoretical sense of the arousal of an elusive and peculiar emotion such as PF in the clinical encounter. When one observes that the clinical encounter has an intercorporeal dimension, an affective interplay between the dynamics of pre-reflective identification, differentiation, and alienness becomes plausible—especially, when one takes alien experience to be something bearing a concrete, though paradoxical, experiential form. On the other hand, we explored the “feel” of PF and aimed to delineate some of its features. The original empirical analysis we performed grasped the two most frequent metaphors employed by physicians to describe PF: “object in-between,” a metaphor for separateness, and “other-worldliness,” a metaphor for strangeness. These two metaphors illuminate the subjective experience of PF, enabling us to show central and seemingly ineffable aspects of PF in concrete imagery, features that until now were only vaguely formulated in previous research.

Along these metaphors and the phenomenological concept of intercorporeal alienness, PF does not lend itself to purely negative formulations: it is not a mere lack of empathy or understanding, but the experience of a multifaceted and unsettling remoteness that appears by getting close to the patient. Some basic components specific to this close remoteness that we propose are 1) a sense of separateness and strangeness, 2) a broken sense of identification, and 3) an eerie feeling that accompanies the intuition of schizophrenia. The phenomenology of PF as intercorporeal alienness has clinical implications on diagnosis, psychopathology, psychotherapy, and clinical training. Last but not least, our current knowledge of this experiential phenomenon has some methodological shortcomings and so far lacks a proper thematization of the patient’s side. The quest to reach a comprehensive understanding of PF is far from ending.

## References

[1] Schwartz MA Wiggins OP. Typifications: the first step for clinical diagnosis in psychiatry. J Nerv Ment Dis. (1987) 175:65–77. doi: 10.1097/00005053-198702000-00001 3806077

[2] Fulford KWM Broome M Stanghellini G Thornton T. Looking with both eyes open: fact and value in psychiatric diagnosis? World Psychiatry Off J World Psychiatr Assoc WPA. (2005) 4:78–86. PMC1414736 16633513

[3] Mann JJ Ellis SP Waternaux CM Liu X Oquendo MA Malone KM. Classification trees distinguish suicide attempters in major psychiatric disorders: a model of clinical decision making. J Clin Psychiatry. (2008) 69:23–31. doi: 10.4088/jcp.v69n0104 18312034 PMC3773877

[4] Pearce S Pickard H. The moral content of psychiatric treatment. Br J Psychiatry. (2009) 195:281–2. doi: 10.1192/bjp.bp.108.062729 19794191

[5] de Vries M Witteman CLM Holland RW Dijksterhuis A. The unconscious thought effect in clinical decision making: an example in diagnosis. Med Decis Making. (2010) 30:578–81. doi: 10.1177/0272989X09360820 20228284

[6] Peters ME Taylor J Lyketsos CG Chisolm MS. Beyond the DSM: the perspectives of psychiatry approach to patients. Prim Care Companion CNS Disord. (2012) 14:26704. doi: 10.4088/PCC.11m01233 PMC3357579 22690367

[7] Lewis B. Taking a narrative turn in psychiatry. Lancet. (2014) 383:22–3. doi: 10.1016/S0140-6736(13)62722-1 24400334

[8] Hoffman GA. Out of our skulls: How the extended mind thesis can extend psychiatry. Philos Psychol. (2016) 29:1160–74. doi: 10.1080/09515089.2016.1236369

[9] Campbell K Massey D Broadbent M Clarke K. Factors influencing clinical decision making used by mental health nurses to provide provisional diagnosis: A scoping review. Int J Ment Health Nurs. (2019) 28:407–24. doi: 10.1111/inm.12553 30394000

[10] Bergqvist A. Narrative understanding, value, and diagnosis: A particularist account of clinical formulations and shared decision-making in mental health. Philos Psychiatry Psychol. (2020) 27:149–67. doi: 10.1353/ppp.2020.0023

[11] Dimic S Wildgrube C McCabe R Hassan I Barnes TRE Priebe S. Non-verbal behaviour of patients with schizophrenia in medical consultations – A comparison with depressed patients and association with symptom levels. Psychopathology. (2010) 43:216–22. doi: 10.1159/000313519 20424502

[12] Yehya A Ghuloum S Mahfoud Z Opler M Khan A Hammoudeh S. Validity and reliability of the arabic version of the positive and negative syndrome scale. Psychopathology. (2016) 49:181–7. doi: 10.1159/000447328 27475457

[13] Blanchard JJ Bradshaw KR Garcia CP Nasrallah HA Harvey PD Casey D. Examining the reliability and validity of the Clinical Assessment Interview for Negative Symptoms within the Management of Schizophrenia in Clinical Practice (MOSAIC) multisite national study. Schizophr Res. (2017) 185:137–43. doi: 10.1016/j.schres.2017.01.011 28087270

[14] Smirnova DA Petrova NN Pavlichenko AV Martynikhin IA Dorofeikova MV Eremkin VI. Multi-centre clinical assessment of the Russian language version of the Diagnostic Interview for Psychoses. Zhurnal Nevrol Psikhiatrii Im SS Korsakova. (2018) 118:50. doi: 10.17116/jnevro20181181150-60 29460905

[15] Tiffin PA Paton LW. The psychometrics of psychosis – assessing and rating perceptual and ideational disturbance in adolescents. Child Adolesc Ment Health. (2019) 24:176–86. doi: 10.1111/camh.12312 32677179

[16] Guinart D de Filippis R Rosson S Patil B Prizgint L Talasazan N. Development and validation of a computerized adaptive assessment tool for discrimination and measurement of psychotic symptoms. Schizophr Bull. (2021) 47:644–52. doi: 10.1093/schbul/sbaa168 PMC8084426 33164091

[17] Lee HS Griffith T Park S. Bodily self-disturbances in schizophrenia: A comparative study of South Korea and the USA. Psychopathology. (2021) 54:262–74. doi: 10.1159/000517933 34380136

[18] Rocha Neto HG Sinem TB Koiller LM Pereira AM de Souza Gomes BM Veloso Filho CL. Intra-rater kappa accuracy of prototype and ICD-10 operational criteria-based diagnoses for mental disorders: A brief report of a cross-sectional study in an outpatient setting. Front Psychiatry. (2022) 13:793743. doi: 10.3389/fpsyt.2022.793743 35308869 PMC8924129

[19] Nishiyama S Kurachi M Higuchi Y Takahashi T Sasabayashi D Mizukami Y. Development and validation of a scale of self-alienation-related attributes for the early diagnosis of schizophrenia. J Psychiatr Res. (2022) 147:212–20. doi: 10.1016/j.jpsychires.2022.01.020 35065511

[20] Misiak B Samochowiec J Kowalski K Gaebel W Bassetti CLA Chan A. The future of diagnosis in clinical neurosciences: Comparing multiple sclerosis and schizophrenia. Eur Psychiatry. (2023) 66:e58. doi: 10.1192/j.eurpsy.2023.2432 37476977 PMC10486256

[21] Parnas J Parnas AU. Refining the diagnostic criteria for schizophrenia: an infinite task. Schizophr Bull. (2024) 50:12–3. doi: 10.1093/schbul/sbad154 PMC10754154 37863120

[22] Urkin B Parnas J Raballo A Koren D. Schizophrenia spectrum disorders: an empirical benchmark study of real-world diagnostic accuracy and reliability among leading international psychiatrists. Schizophr Bull Open. (2024) 5:sgae012. doi: 10.1093/schizbullopen/sgae012 39144107 PMC11207759

[23] Picardi A Panunzi S Misuraca S Di Maggio C Maugeri A Fonzi L. The clinician’s subjective experience during the interaction with adolescent psychiatric patients: validity and reliability of the assessment of clinician’s subjective experience. Psychopathology. (2021) 54:119–26. doi: 10.1159/000513769 33789281

[24] Tellenbach H. Geschmack und atmosphäre. Salzburg: O. Muller. (1968) p. 175.

[25] Carp EADE. Über das praecox-und hysteriegefühl. Psychother Psychosom. (1971) 19:232–9. doi: 10.1159/000286321 5149833

[26] Kraus A Maj M Sartorious N. The significance of intuition for the diagnosis of schizophrenia. In: Schizophrenia. John Wiley & Sons, Chichester, UK (1999). p. 47–9.

[27] Parnas J. A disappearing heritage: the clinical core of schizophrenia. Schizophr Bull. (2011) 37:1121–30. doi: 10.1093/schbul/sbr081 PMC3196960 21771902

[28] Buonarroti M Fonzi L Pallagrosi M. The clinician’s subjective feeling in psychiatric diagnosis: A historical excursus. In: Biondi M Picardi A Pallagrosi M Fonzi L, editors. The clinician in the psychiatric diagnostic process, Springer International Publishing, Cham (2022). p. 1–24. doi: 10.1007/978-3-030-90431-9_1

[29] Fuchs T Dalpane E. The psychiatric assessment: first person, second person, and third person perspectives. In: Biondi M Picardi A Pallagrosi M Fonzi L, editors. The clinician in the psychiatric diagnostic process. Springer International Publishing, Cham (2022). p. 25–36. doi: 10.1007/978-3-030-90431-9_2

[30] Moskalewicz M Gozé T. Clinical judgment of schizophrenia: praecox feeling and the bizarreness of contact—Open controversies. In: Biondi M Picardi A Pallagrosi M Fonzi L, editors. The clinician in the psychiatric diagnostic process. Springer International Publishing, Cham (2022). p. 135–49. doi: 10.1007/978-3-030-90431-9_9

[31] Zaninotto L Altobrando A. Four core concepts in psychiatric diagnosis. Psychopathology. (2022) 55:73–81. doi: 10.1159/000520334 34965532

[32] Bizzari V Brencio F. Psychiatric diagnosis as a political and social device: Epistemological and historical insights on the role of collective emotions. Humanist Psychol. (2024) 52:70–82. doi: 10.1037/hum0000307

[33] Rümke HC. Das Kernsymptom der Schizophrenie und das ‘Praecox Gefühl. ’ Z Gesamte Neurol Psychiatr. (1941) 102:168–75.

[34] Rümke HC Neeleman J. The nuclear symptom of schizophrenia and the praecoxfeeling. Hist Psychiatry. (1990) 1:331–41. doi: 10.1177/0957154X9000100304 11622458

[35] Binswanger L. Welche Aufgaben ergeben sick für die Psychiatrie aus den Fortschritten der neueren Psychologie? Z Für Gesamte Neurol Psychiatr. (1924) 91:402–36. doi: 10.1007/BF02877892

[36] Minkowski E. La schizophrénie. Paris: Payot (1927).

[37] Pallagrosi M Fonzi L. On the concept of praecox feeling. Psychopathology. (2018) 51:353–61. doi: 10.1159/000494088 30380532

[38] Grube M. Towards an empirically based validation of intuitive diagnostic: rümke’s ‘Praecox feeling’ across the schizophrenia spectrum: preliminary results. Psychopathology. (2006) 39:209–17. doi: 10.1159/000093921 16778451

[39] Ungvari GS Xiang YT Hong Y Leung HCM Chiu HFK. Diagnosis of schizophrenia: reliability of an operationalized approach to ‘Praecox-feeling. ’ Psychopathol. (2010) 43:292–9. doi: 10.1159/000318813 20639689

[40] Gozé T Moskalewicz M Schwartz MA Naudin J Micoulaud-Franchi JA Cermolacce M. Is “praecox feeling” a phenomenological fossil? A preliminary study on diagnostic decision making in schizophrenia. Schizophr Res. (2019) 204:413–4. doi: 10.1016/j.schres.2018.07.041 30072280

[41] Gozé T Moskalewicz M Schwartz MA Naudin J Micoulaud-Franchi JA Cermolacce M. Reassessing “Praecox feeling” in diagnostic decision making in schizophrenia: A critical review. Schizophr Bull. (2019) 45:966–70. doi: 10.1093/schbul/sby172 PMC6737542 30476340

[42] Moskalewicz M Kordel P Brejwo A Schwartz MA Gozé T. Psychiatrists report praecox feeling and find it reliable. A Cross-Cultural Comparison. Front Psychiatry. (2021) 12:642322. doi: 10.3389/fpsyt.2021.642322 33746799 PMC7973011

[43] Varga S. Vulnerability to psychosis, I-thou intersubjectivity and the praecox-feeling. Phenomenol Cognit Sci. (2013) 12:131–43. doi: 10.1007/s11097-010-9173-z

[44] Moskalewicz M Schwartz MA Gozé T. Phenomenology of intuitive judgment: praecox-feeling in the diagnosis of schizophrenia. AVANT J Philos-Interdiscip Vanguard. (2018) 9:63–74. doi: 10.26913/avant.2018.02.04

[45] Merleau-Ponty M. The philosopher and his shadow. In: Signs. Northwestern University Press, Evanston (1964). p. 159–81.

[46] Merleau-Ponty M. The child’s relations with others. In: The primacy of perception. Northwestern University Press, Evanston (1964). p. 96–155.

[47] Fuchs T. Leibliche Kommunikation und ihre Störungen. ZKPPP. (1996) 44:415–28.

[48] Fuchs TL. Leib, Raum, Person. Entwurf einer phänomenologischen Anthropologie. Stuttgart: Klett-Cotta (2000). 419 p.

[49] Waldenfels B. Das leibliche Selbst: Vorlesungen zur Phänomenologie des Leibes. Frankfurt am Main: Suhrkamp (2000). 417 p.

[50] Fuchs T Koch SC. Embodied affectivity: on moving and being moved. Front Psychol. (2014) 5:508. doi: 10.3389/fpsyg.2014.00508 24936191 PMC4047516

[51] Fuchs T. Intercorporeality and interaffectivity. In: Meyer C Streeck J Jordan JS, editors. Intercorporeality: Emerging socialities in interaction. Oxford Univesity Press, Oxford (2017). p. 3–23.

[52] Fuchs T. Zwischenleibliche diagnostik. In: Höfner C Hochgerner M, editors. Psychotherapeutische diagnostik. Springer Berlin Heidelberg, Berlin, Heidelberg (2022). p. 63–73. doi: 10.1007/978-3-662-61450-1_5

[53] Waldenfels B. Bodily experience between selfhood and otherness. Phenomenol Cognit Sci. (2004) 3:235–48. doi: 10.1023/B:PHEN.0000049305.92374.0b

[54] Moran D. Intercorporeality and intersubjectivity: A phenomenological exploration of embodiment. In: Durt C Fuchs T Tewes C, editors. Embodiment, enaction, and culture. Cambridge, MA: The MIT Press (2017). p. 25–47. doi: 10.7551/mitpress/10799.003.0004

[55] Husserl E. Cartesian meditations. An introduction to phenomenology. Martinus Nijhoff, The Hage (1960).

[56] León F Szanto T Zahavi D. Emotional sharing and the extended mind. Synthese. (2019) 196:4847–67. doi: 10.1007/s11229-017-1351-x

[57] Lévinas E. Totality and infinity. Dordrecht: Springer Netherlands (1991). 297 p. doi: 10.1007/978-94-009-9342-6

[58] Zahavi D. Self-awareness and alterity: a phenomenological investigation. Evanston, Ill: Northwestern University Press (1999) 291 p.

[59] Waldenfels B. Phenomenology of the alien: basic concepts. Evanston, Ill: Northwestern University Press (2011) 91 p. doi: 10.2307/j.ctv47wfh3

[60] Gozé T. Expérience de la rencontre schizophrénique: de la bizarrerie de contact. Paris: Hermann. (2020) p. 254.

[61] Sass L Feyaerts J. Schizophrenia, the very idea: On self-disorder, hyperreflexivity, and the diagnostic concept. Schizophr Res. (2024) 267:473–86. doi: 10.1016/j.schres.2024.03.022 38693032

[62] Kristeva J. Strangers to ourselves. New York: Columbia Univ. Press (1991). 230 p.

[63] Waldenfels B. Topographie des fremden. In: Studien zur Phänomenologie des Fremden, vol. 1. Suhrkamp, Frankfurt am Main (1997). p. 227 p.

[64] Waldenfels B. Experience of the alien in husserl’s phenomenology. Res Phenomenol. (1990) 20:19–33.

[65] Waldenfels B. Bruchlinien der erfahrung: phänomenologie, psychoanalyse, phänomenotechnik. Frankfurt am Main: Suhrkamp (2002). 478 p.

[66] Hempel CG. Aspects of scientific explanation. New York: Free Press (1965).

[67] Waldenfels B. The role of the lived-body in feeling. Cont Philos Rev. (2008) 41:127–42. doi: 10.1007/s11007-008-9077-6

[68] Sagi GA Schwartz MA. The “praecox feeling” in the diagnosis of schizophrenia; a survey of Manhattan psychiatrists. Schizophr Res. (1989) 2:35. doi: 10.1016/0920-9964(89)90071-6

[69] Szuła A Kołtun A Moskalewicz M. Praecox feeling among Polish psychiatrists: a grounded theory study. Psychiatr Pol. (2023) 325:1–15. doi: 10.12740/PP/OnlineFirst/166599 38055897

[70] Gozé T. How to teach/learn praecox feeling? Through phenomenology to medical education. Front Psychiatry. (2022) 13:819305. doi: 10.3389/fpsyt.2022.819305 35370862 PMC8971516

[71] Arendt H. The life of the mind. Thinking. Vol. 1. New York: Harcourt Brace Jovanovich. (1978) p. 258.

[72] Cornejo C. Review Essay: Conceptualizing Metaphors versus Embodying the Language: Kövecses, Zoltán, Metaphor in Culture: Universality and Variation. Cambridge: Cambridge Universty Press. (2007) 13:474–87. doi: 10.1177/1354067X07082806. (hbk). Cult Psychol.

[73] Cornejo C Olivares H Rojas P. The physiognomic and the geometrical apprehensions of metaphor. Cult Psychol. (2013) 19:484–505. doi: 10.1177/1354067X13500330

[74] Kövecses Z. American friendship and the scope of metaphor. Cognit Linguist. (1995) 6:315–46. doi: 10.1515/cogl.1995.6.4.315

[75] Steen G. When is metaphor deliberate? In: Johannesson NL Alm-Arvius C Minugh DC, editors. Selected papers from the stockholm 2008 metaphor festival. University of Stokcholm, Stokcholm (2011). p. 43–63.

[76] Fuchs T. The interactive phenomenal field and the life space: A sketch of an ecological concept of psychotherapy. Psychopathology. (2019) 52:67–74. doi: 10.1159/000502098 31394534

[77] Heidegger M. Being and time. Oxford: Blackwell (1962). 589 p.

[78] Jaspers K. General psychopathology. 7th ed. Manchester: Manchester Univ. Press (1967). 922 p.

[79] Fuchs T. Pathologies of intersubjectivity in autism and schizophrenia. J Conscious Stud. (2015) 22:191–214.

[80] Wendler H Fuchs T. Understanding incomprehensibility: Misgivings and potentials of the phenomenological psychopathology of schizophrenia. Front Psychol. (2023) 14:1155838. doi: 10.3389/fpsyg.2023.1155838 37057153 PMC10086155

[81] Husserl E. Zur Phänomenologie der Intersubjektivität III. Texte aus dem Nachlass. Dritter teil. 1929–1935. Kern I, editor. The Hage: Martinus Nijhoff (1973). 741 p.

[82] Fuchs T De Jaegher H. Enactive intersubjectivity: Participatory sense-making and mutual incorporation. Phenom Cognit Sci. (2009) 8:465–86. doi: 10.1007/s11097-009-9136-4

[83] Tschacher W Giersch A Friston K. Embodiment and schizophrenia: A review of implications and applications. Schizophr Bull. (2017) 43:745–53. doi: 10.1093/schbul/sbw220 PMC5472128 28338892

[84] Cornejo C Hurtado E Cuadros Z Torres-Araneda A Paredes J Olivares H. Dynamics of simultaneous and imitative bodily coordination in trust and distrust. Front Psychol. (2018) 9:1546. doi: 10.3389/fpsyg.2018.01546 30210391 PMC6121516

[85] Ellgring H. Nonverbal expression of psychological states in psychiatric patients. Eur Arch Psychiatry Neurol Sci. (1986) 236:31–4. doi: 10.1007/BF00641055 3743583

[86] Varlet M Marin L Raffard S Schmidt RC Capdevielle D Boulenger JP. Impairments of social motor coordination in schizophrenia. PloS One. (2012) 7:e29772. doi: 10.1371/journal.pone.0029772 22272247 PMC3260163

[87] Lavelle M Healey PG McCabe R. Is nonverbal communication disrupted in interactions involving patients with schizophrenia? Schizophr Bull. (2013) 39:1150–8. doi: 10.1093/schbul/sbs091 PMC3756773 22941744

[88] Matthews N Gold BJ Sekuler R Park S. Gesture imitation in schizophrenia. Schizophr Bull. (2013) 39:94–101. doi: 10.1093/schbul/sbr062 21765171 PMC3523902

[89] Kupper Z Ramseyer F Hoffmann H Tschacher W. Nonverbal synchrony in social interactions of patients with schizophrenia indicates socio-communicative deficits. PloS One. (2015) 10:e0145882. doi: 10.1371/journal.pone.0145882 26716444 PMC4696745

[90] Słowiński P Alderisio F Zhai C Shen Y Tino P Bortolon C. Unravelling sociomotor biomarkers in schizophrenia. NPJ Schizophr. (2017) 3:8. doi: 10.1038/s41537-016-0009-x 28560254 PMC5441525

[91] Dean DJ Scott J Park S. Interpersonal coordination in schizophrenia: A scoping review of the literature. Schizophr Bull. (2021) 47:1544–56. doi: 10.1093/schbul/sbab072 PMC8530389 34132344

[92] Pan Y Wen Y Wang Y Schilbach L Chen J. Interpersonal coordination in schizophrenia: a concise update on paradigms, computations, and neuroimaging findings. Psychoradiology. (2023) 3:kkad002. doi: 10.1093/psyrad/kkad002 38666124 PMC10917372

[93] Tahir Y Yang Z Chakraborty D Thalmann N Thalmann D Maniam Y. Non-verbal speech cues as objective measures for negative symptoms in patients with schizophrenia. PloS One. (2019) 14:e0214314. doi: 10.1371/journal.pone.0214314 30964869 PMC6456189

[94] Lucarini V Grice M Wehrle S Cangemi F Giustozzi F Amorosi S. Language in the interaction: turn-taking patterns in conversations involving individuals with schizophrenia. Psychiatry Res. (2024) 339:116102. doi: 10.1016/j.psychres.2024.116102 39089189

[95] Hatfield E Cacioppo JT Rapson RL. Emotional contagion. Cambridge: Cambridge Univ. Press (1994). 240 p.

[96] Hatfield E Carpenter M Rapson RL. Emotional contagion as a precursor to collective emotions. In: von Scheve C Salmela M, editors. Collective emotions. Oxford: Oxford University Press (2014). p. 108–22. doi: 10.1093/acprof:oso/9780199659180.003.0008

[97] Haker H Rössler W. Empathy in schizophrenia: impaired resonance. Eur Arch Psychiatry Clin Neurosci. (2009) 259:352–61. doi: 10.1007/s00406-009-0007-3 19377866

[98] Kring AM Kerr SL Smith DA Neale JM. Flat affect in schizophrenia does not reflect diminished subjective experience of emotion. J Abnorm Psychol. (1993) 102:507–17. doi: 10.1037/0021-843X.102.4.507 8282918

[99] Mattes RM Schneider F Heimann H Birbaumer N. Reduced emotional response of schizophrenic patients in remission during social interaction. Schizophr Res. (1995) 17:249–55. doi: 10.1016/0920-9964(95)00014-3 8664204

[100] Mandal MK Pandey R Prasad AB. Facial expressions of emotions and schizophrenia: A review. Schizophr Bull. (1998) 24:399 412. doi: 10.1093/oxfordjournals.schbul.a033335 9718632

[101] Gaebel W Wölwer W. Facial expressivity in the course of schizophrenia and depression. Eur Arch Psychiat Clin Neurosci. (2004) 254:335–42. doi: 10.1007/s00406-004-0510-5 15365710

[102] Trémeau F. A review of emotion deficits in schizophrenia. DCNS. (2006) 8:59–70. doi: 10.31887/DCNS.2006.8.1/ftremeau PMC3181757 16640115

[103] Kohler CG Martin EA Stolar N Barrett FS Verma R Brensinger C. Static posed and evoked facial expressions of emotions in schizophrenia. Schizophr Res. (2008) 105:49–60. doi: 10.1016/j.schres.2008.05.010 18789845 PMC5048468

[104] Lavelle M Healey PG McCabe R. Nonverbal behavior during face-to-face social interaction in schizophrenia: a review. J Nerv Ment Dis. (2014) 202:47–54. doi: 10.1097/NMD.0000000000000031 24375212

[105] Troisi A Pasini A Bersani M Di Mauro M Clani N. Negative symptoms and visual behavior in DSM-III-R prognostic subtypes of schizophreniform disorder. Acta Psychiatr Scand. (1991) 83:391–4. doi: 10.1111/j.1600-0447.1991.tb05562.x 1853733

[106] Davison PS Frith CD Harrison-Read PE Johnstone EC. Facial and other non-verbal communicative behaviour in chronic schizophrenia. Psychol Med. (1996) 26:707–13. doi: 10.1017/S0033291700037727 8817705

[107] Choi SH Ku J Han K Kim E Kim SI Park J. Deficits in eye gaze during negative social interactions in patients with schizophrenia. J Nerv Ment Des. (2010) 198:829–35. doi: 10.1097/NMD.0b013e3181f97c0d 21048475

[108] Vail AK Baltrušaitis T Pennant L Liebson E Baker J Morency LP. Visual attention in schizophrenia: Eye contact and gaze aversion during clinical interactions. In: 2017 Seventh International Conference on Affective Computing and Intelligent Interaction (ACII). (San Antonio, TX: IEEE). (2017) p. 490–7. doi: 10.1109/ACII.2017.8273644

[109] Rümke HC. Eine blühende psychiatrie in gefahr. Ritter von Baeyer W, editor. Berlin, Heidelberg: Springer Berlin Heidelberg (1967). doi: 10.1007/978-3-642-86491-9

[110] Waldenfels B. Der stachel des fremden. Frankfurt am Main: Suhrkamp (1990). 278 p.

[111] Fisher M. The weird and the eerie. London: Repeater Books (2016). 133p.

[112] Fuchs T. The phenomenology of body memory. In: Koch S Müller C Summa M Fuchs T, editors. Body memory, metaphor, and movement. John Benjamins, Amsterdam (2012). p. 9–22.

[113] Pallagrosi M Fonzi L Picardi A Biondi M. Assessing clinician's subjective experience during interaction with patients. Psychopathology. (2014) 47:111–8. doi: 10.1159/000351589 23942272

[114] Pallagrosi M Fonzi L Picardi A Biondi M. Association between clinician’s subjective experience during patient evaluation and psychiatric diagnosis. Psychopathology. (2016) 49:83–94. doi: 10.1159/000444506 27073874

[115] Picardi A Pallagrosi M Fonzi L Biondi M. Psychopathological dimensions and the clinician’s subjective experience. Psychiatry Res. (2017) 258:407–14. doi: 10.1016/j.psychres.2017.08.079 28870646

[116] Galbusera L Fellin L. The intersubjective endeavor of psychopathology research: methodological reflections on a second-person perspective approach. Front Psychol. (2014) 5:1150. doi: 10.3389/fpsyg.2014.01150 25368589 PMC4201097

[117] Schilbach L. Towards a second-person neuropsychiatry. Phil Trans R Soc B. (2016) 371:20150081. doi: 10.1098/rstb.2015.0081 26644599 PMC4685526

[118] León F Zandersen M Meindl P Zahavi D. The distinction between second-person and third-person relations and its relevance for the psychiatric diagnostic interview. In: Biondi M Picardi A Pallagrosi M Fonzi L, editors. The clinician in the psychiatric diagnostic process. Springer International Publishing, Cham (2022). p. 51–69. doi: 10.1007/978-3-030-90431-9_4

[119] Petitmengin C. Describing one’s subjective experience in the second person: An interview method for the science of consciousness. Phenomenol Cognit Sci. (2006) 5:229–69. doi: 10.1007/s11097-006-9022-2

[120] Sass L. Affectivity in schizophrenia a phenomenological view. J Conscious Stud. (2004) 11:127–47.

[121] Stephensen H Parnas J. What can self-disorders in schizophrenia tell us about the nature of subjectivity? A psychopathological investigation. Phenomenol Cognit Sci. (2018 2018) 17:629–42. doi: 10.1007/s11097-017-9532-0

[122] Parnas J Urfer-Parnas A Stephensen H. Double bookkeeping and schizophrenia spectrum: divided unified phenomenal consciousness. Eur Arch Psychiatry Clin Neurosci. (2021) 271:1513–23. doi: 10.1007/s00406-020-01185-0 PMC8563555 32901298

[123] Stephensen H Urfer-Parnas A Parnas J. Double bookkeeping in schizophrenia spectrum disorder: an empirical-phenomenological study. Eur Arch Psychiatry Clin Neurosci. (2023) 274:1405–15. doi: 10.1007/s00406-023-01609-7 PMC11362502 37084145

